# Molecular profiling of male breast cancer by multigene panel testing: Implications for precision oncology

**DOI:** 10.3389/fonc.2022.1092201

**Published:** 2023-01-06

**Authors:** Virginia Valentini, Valentina Silvestri, Agostino Bucalo, Giulia Conti, Mina Karimi, Linda Di Francesco, Giulia Pomati, Silvia Mezi, Bruna Cerbelli, Maria Gemma Pignataro, Arianna Nicolussi, Anna Coppa, Giulia D’Amati, Giuseppe Giannini, Laura Ottini

**Affiliations:** ^1^ Department of Molecular Medicine, Sapienza University of Rome, Rome, Italy; ^2^ Department of Radiological, Oncological and Pathological Sciences, Sapienza University of Rome, Rome, Italy; ^3^ Department of Medical-Surgical Sciences and Biotechnologies Sapienza University of Rome, Rome, Italy; ^4^ Department of Experimental Medicine, Sapienza University of Rome, Rome, Italy; ^5^ Istituto Pasteur-Fondazione Cenci Bolognetti, Rome, Italy

**Keywords:** male breast cancer (MBC), tumor profiling, targeted gene panel sequencing, clinically actionable genetic variants, tumor mutational burden (TMB), microsatellite instability (MSI), copy number variations (CNVs), precision oncology

## Abstract

**Introduction:**

Compared with breast cancer (BC) in women, BC in men is a rare disease with genetic and molecular peculiarities. Therapeutic approaches for male BC (MBC) are currently extrapolated from the clinical management of female BC, although the disease does not exactly overlap in males and females. Data on specific molecular biomarkers in MBC are lacking, cutting out male patients from more appropriate therapeutic strategies. Growing evidence indicates that Next Generation Sequencing (NGS) multigene panel testing can be used for the detection of predictive molecular biomarkers, including Tumor Mutational Burden (TMB) and Microsatellite Instability (MSI).

**Methods:**

In this study, NGS multigene gene panel sequencing, targeting 1.94 Mb of the genome at 523 cancer-relevant genes (TruSight Oncology 500, Illumina), was used to identify and characterize somatic variants, Copy Number Variations (CNVs), TMB and MSI, in 15 Formalin-Fixed Paraffin-Embedded (FFPE) male breast cancer samples.

**Results and discussion:**

A total of 40 pathogenic variants were detected in 24 genes. All MBC cases harbored at least one pathogenic variant. *PIK3CA* was the most frequently mutated gene, with six (40.0%) MBCs harboring targetable *PIK3CA* alterations. CNVs analysis showed copy number gains in 22 genes. No copy number losses were found. Specifically, 13 (86.7%) MBCs showed gene copy number gains. *MYC* was the most frequently amplified gene with eight (53.3%) MBCs showing a median fold-changes value of 1.9 (range 1.8-3.8). A median TMB value of 4.3 (range 0.8-12.3) mut/Mb was observed, with two (13%) MBCs showing high-TMB. The median percentage of MSI was 2.4% (range 0-17.6%), with two (13%) MBCs showing high-MSI. Overall, these results indicate that NGS multigene panel sequencing can provide a comprehensive molecular tumor profiling in MBC. The identification of targetable molecular alterations in more than 70% of MBCs suggests that the NGS approach may allow for the selection of MBC patients eligible for precision/targeted therapy.

## Introduction

Male breast cancer (MBC) is a rare disease representing less than 1.0% of all breast cancers (BCs) and less than 1.0% of all cancers in men (1). Despite its rarity, the annual incidence of MBC continues to arise and is estimated at about 1 per 100.000 men (2).

Increasing evidence indicates that MBC and female breast cancer (FBC) may be different, with unique molecular subtypes suggesting gender-specific differences in terms of biological and clinical behavior (3). Despite distinct features, therapeutic approaches for MBC are extrapolated from clinical management guidelines relating to FBC (4). Overall, MBC has a poorer outcome, likely due to its occurrence later in life, the delay in diagnosis compared with the female counterpart (5), or to gender-specific factors yet to be identified.

The development of Next Generation Sequencing (NGS) technologies has produced a large amount of research data about genomic alterations in a wide variety of cancers, including BC (6). These large-scale initiatives have identified genomic alterations that are potential therapeutic targets to guide individualized treatment (7). Actionable genomic alterations include genetic variants, Copy Number Variations (CNVs), Tumor Mutational Burden (TMB) and Microsatellite Instability (MSI). Tumor-infiltrating lymphocytes (TILs) have also been suggested to represent potentially useful prognostic and predictive biomarkers, especially in triple negative FBCs (8).

While therapies based on NGS findings are a new standard of care for treating a variety of cancers, only a few studies have been performed to comprehensively characterize tumor profiles in MBCs (9–12) and data on specific molecular biomarkers in MBC are lacking. Moreover, associations among new potential molecular biomarkers and clinical data remain unclear in MBC, cutting out male patients from new targeted treatments. Thus, there is a need to investigate molecular biomarkers predictive of response to innovative treatments for a more effective clinical management of MBC patients.

In this pilot study, we characterized the molecular tumor profiling of 15 MBCs by targeted gene panel sequencing to identify clinically actionable somatic variants, CNVs, TMB and MSI status that may represent new predictive biomarkers for MBC patients.

## Material and methods

### Study population

This is an observational, retrospective study, based on a series of 15 MBC cases collected between January 2012 and December 2021 at our Institution.

All patients enrolled had been previously tested for germline pathogenic variants in BC predisposition genes including *BRCA1*, *BRCA2*, and *PALB2*. The main clinical-pathologic features, including age at diagnosis, tumor histotype, histologic grade, nodal status, Estrogen/Progesterone Receptor (ER, PR), Androgen Receptor (AR), Human Epidermal growth factor Receptor 2 (HER2) and proliferation index (Ki67/MIB1) status were collected. Cases were classified as Luminal A-like; Luminal B-like (HER2 negative or positive), HER2-enriched and triple negative, according to the 13th St. Gallen International Breast Cancer Conference (13).

MBC cases were all primary cancer with the exception of one case for which only samples from pleural metastases were available.

All patients signed an informed consent form with a detailed description of the study protocol. The study was approved by The Local Ethical Committee (Sapienza University of Rome, Protocol 669/17) and was performed according to the Helsinki’s declaration.

### Quantification and characterization of stromal tumor–infiltrating lymphocytes

Haematoxylin and eosin-stained slides from all cases were re-evaluated by two breast pathologists (BC, GdA) for the presence and percentage of stromal tumor infiltrating lymphocytes (TILs), according to the standardized method proposed by the International TILs Working Group in 2014 (8). TILs were quantified as a percentage of the stromal area of the tumor and expressed as a continuous parameter. Cases were stratified into high- and low- TILs according to the cut-off of 50% (8). Immunophenotyping was carried out on serial sections from each case with the following antibodies: CD3 for T lymphocytes (1: 100 Roche Diagnostics, Basilea, Switzerland); CD4 (1: 40) for the helper T subset; CD8 for the cytotoxic T subset (1: 100) (Novocastra, Newcastle, UK). The number of lymphocytes positive for each antibody was quantified and expressed as a percentage of the total number of immune cells.

### DNA extraction

Genomic DNA was extracted from 10 μm-thick macroscopically dissected formalin fixed paraffin-embedded (FFPE) tumor sections. For each case, a representative haematoxylin and eosin-stained slide was obtained and marked for an area with high tumor cellularity, to obtain a tumor cell percentage ranging from 50% to 90%. To avoid pitfalls related to the use of archival materials, freshly cut sections and specific extraction protocols (14), developed to improve DNA quality and quantity from FFPE samples, were used. DNA was extracted using the QIAamp DNA FFPE tissue kit (Qiagen, Hilden, Germany), according to the manufacturer’s instructions. Eluted DNA was quantified with Qubit 2.0 Fluorometer using the Qubit dsDNA HS Assay Kit (ThermoFisher Scientific, Waltham, Massachusetts, USA). The suitability of samples for sequencing was determined using the real-time PCR-based Illumina FFPE QC assay (Illumina, San Diego, California, USA), as detailed in the **Supplementary material**.

### TruSight oncology 500 sequencing and variant calling

TruSight Oncology 500 is a targeted gene panel that covers 1.94 Mb of the genome in 523 cancer-relevant genes (**Supplementary Table 1**). DNA libraries were prepared using the hybrid capture based TruSight Oncology 500 Library Preparation Kit (Illumina, San Diego, California, USA) following Illumina’s TruSight Oncology 500 Reference Guide. The enriched libraries were quantified, and each library was normalized to ensure a uniform representation in the pooled libraries. Finally, the libraries were denatured and diluted to the appropriate loading concentration. The libraries were sequenced on an Illumina NextSeq 500 instrument, with a read length of 2x101 bp, and up to 8 libraries per run, according to the manufacturer’s protocols.

Sequencing data and DNA quality metrics are reported in the **Supplemental Material**.

Files containing reads (.fastq) were generated and processed by alignment against the human reference genome GRCh37/hg19 using the Burrows-Wheeler Aligner (BWA-MEM) with the SAM Tools. Pisces application was applied to performing somatic variant calling. CNV calls (gain and losses) were obtained for 59 out of 523 genes within the TruSight Oncology 500 panel (**Supplementary Table 1**), using the Craft copy-number caller. The resulting variant and CNV calling files (.vcf) were processed on BaseSpace Variant Interpreter (Illumina, https://variantinterpreter.informatics.illumina.com) and Open-CRAVAT (https://opencravat.org), for variant annotation and classification. MSI calls were generated by analyzing 125 homopolymeric microsatellite loci with a coverage of at least 60 reads. MSI values were obtained by dividing the number of unstable sites by the total number of sites assessed defining the percentage of unstable sites.

### Characterization of the somatic molecular profile

For the subsequent analysis, we considered all variants and CNVs that passed the quality filters, marked as PASS in the output files of variant annotation and classification step (**Supplementary Table 2**).

To identify the pathogenic (driver) somatic variants, all PASS variants were filtered to include: exonic (except for synonymous variants) or splice site variants with an allelic frequency between 5.0% and 90.0%, a total read depth ≥40, a global Allele Frequency < 0.01 in the gnomAD population database; classified as somatic according to Cancer Gene Census (CGC) database and as pathogenic according to the Catalogue of Somatic Mutations in Cancer (COSMIC) database.

Moreover, in MBC cases tested positive for a *BRCA1, BRCA2* or *PALB2* germline pathogenic variant, the loss of wild-type allele in tumoral sample was evaluated. Germline pathogenic variants with an allelic frequency greater than 50.0% were considered as loss of heterozygosity (LOH) and subsequently validated by Sanger sequencing (primers available upon request).

CNVs were considered as gains with a fold-change value ≥ 1.5 (three copies) and as losses with a fold-change value ≤ 0.5 (one copy).

The panel size allowed for the characterization of TMB status in MBC samples. An in-house developed pipeline was used to select the eligible variants for TMB calculation (manuscript in preparation). Tumor samples were stratified as high- and low-TMB by the conventional cut-off value of 10 mutations/Megabase (mut/Mb) (15, 16).

Similarly, tumor samples were stratified as high- and low-MSI by the previously established percentage of unstable sites ≥10%, as an identifier of presumed microsatellite instability (17). MBC cases were classified as stable if they resulted to have no unstable sites.

We then sought to determine if the identified alterations could translate into actionable targets with possible clinical implications in BC, by interrogating the OncoKB database (https://www.oncokb.org/), a comprehensive and curated precision oncology knowledge base that provides biological, clinical, and therapeutical information.

## Results

### Clinical-pathologic characteristics of MBCs

**Table 1** summarizes the clinical-pathologic characteristics of the 15 MBC cases included in this study. Briefly, the MBC series included four cases (26.7%) with germline pathogenic variants in *BRCA1* (two), *BRCA2* (one) and *PALB2* (one) genes, mean age at BC diagnosis was 63.0 years (range 36-88 years) and median follow-up was 3.0 years (range 1-10 years). Four MBC cases (28.6%) had breast and ovarian cancer family history and two MBC cases (13.3%) were diagnosed with another cancer, in addition to BC.

**Table 1:** Clinical-pathologic characteristics of the 15 MBCs analyzed in this study.

Characteristic	No.	%
Genetic testing		
*BRCA1/2* and *PALB2* negative	11	73.3
*BRCA1/2* and *PALB2* positive	4	26.7
**Median age at diagnosis ± SD (range)**	63 ± 12.8 (36-88)	
**Median follow-up, years ± SD (range)**	3 ± 2.7 (1-10)	
First-degree family history of BC/OC*		
Negative	10	71.4
Positive	4	28.6
Personal history of cancer in addition to BC		
Negative	13	86.7
Positive	2	13.3
Tumor histotype		
Invasive carcinoma, NST	15	100.0
Pathological TNM stage *		
I	6	42.9
II	3	21.4
III	5	35.7
IV	0	0.0
Histologic grade		
1	0	0.0
2	7	46.7
3	8	53.3
Lymph node status*		
Negative	7	50.0
Positive	7	50.0
ER status		
Negative	2	13.3
Positive	13	86.7
PR status		
Negative	1	6.7
Positive	14	93.3
AR status*		
Negative	0	0.0
Positive	14	100.0
HER2 status*		
Negative	11	78.6
Positive	3	21.4
Ki67/MIB1 status		
Low	4	26.7
High	11	73.3
Intrinsic subtype*		
Luminal-A	3	21.4
Luminal-B/HER2 negative	8	57.2
Luminal-B/HER2 positive	2	14.3
HER2-enriched	1	7.1
Tumor-infiltrating lymphocytes*		
Negative	2	14.3
Low TILs	11	78.6
High TILs	1	7.1

*For one case this characteristic is not available.

SD, Standard Deviation; BC, Breast Cancer; OC, Ovarian Cancer; NST, no special type.

As shown in **Table 1**, all tumors were classified as invasive carcinoma of no special type (NST), with histologic grade 2 (intermediate) and 3 (high) in 46.7% and 53.3% of MBCs, respectively. At diagnosis, nine of the 15 cases were referred to I/II TNM stage (64.3%) and five were referred to III TNM stage (35.7%); for one case TNM stage was not available. The case for which only samples from pleural metastases were analyzed, developed metastases during the follow-up period.

As expected, most of MBCs were ER positive (86.7%), PR positive (93.3%) and HER2 negative (78.6%). High AR expression was observed in all MBCs for which this information was available.

There was a prevalence (73.3%) of cases with high proliferation rate at diagnosis. Overall, most cases showed a Luminal-B intrinsic subtype (57.2% were HER2 negative and 14.3% were HER2 positive) and 21.4% showed a Luminal-A intrinsic subtype. Only one case (7.1%) showed a HER2-enriched intrinsic subtype. The range of TILs was 0-70%, with a median value of 10%. The vast majority of MBCs (92.9%) had negative/low TILs.

### Somatic variants, CNV, TMB and MSI by NGS

Somatic pathogenic variants were identified in 24 of the 523 genes included in the panel. Overall, 40 somatic pathogenic variants were detected and included 35 (87.5%) missense variants, three (7.5%) frameshift deletions and two (5.0%) stop-gained variants (**Supplementary Table 3**).

**Figure 1:**
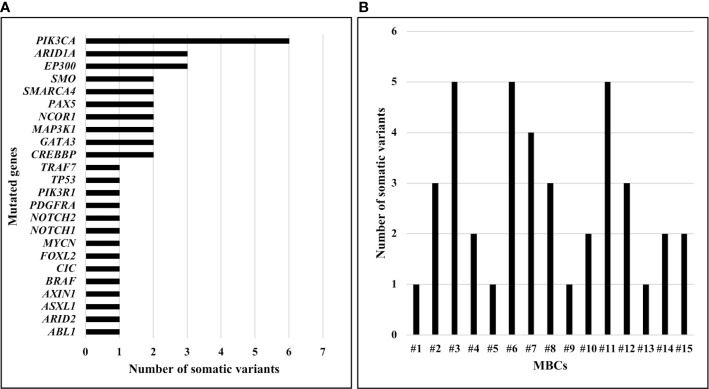
Number of somatic pathogenic variants identified in MBCs using TruSight Oncology 500. **(A)** Number of somatic pathogenic variants identified, by gene. **(B)** Number of somatic pathogenic variants identified, by sample.

*PIK3CA* was the most frequently mutated gene. Specifically, *PIK3CA* variants were detected in six (40.0%) MBCs (**Figure 1A**). Somatic variants in *ARID1A* and *EP300* were identified in three (20.0%) MBCs, in *SMO*, *SMARCA4, PAX5, NCOR1, MAP3K1, GATA3, CREBBP* in two (13.3%) MBCs, each and in *TRAF7, TP53, PIK3R1, PDGFRA, NOTCH2, NOTCH1, FOXL2, CIC, BRAF, AXIN1, ASXL1, ARID2, ABL1* in one (6.7%) MBC, each (**Figure 1A**). A median of two (range 1-5) somatic variants per case was observed, with three (20.0%) MBCs showing five somatic variants, each (**Figure 1B**).

The loss of the wild-type allele was identified in two out of four MBCs with germline pathogenic variant, specifically, in *BRCA1* and *PALB2* carriers (**Table 2**).

**Table 2:** Actionable molecular alterations identified in the 15 MBCs analyzed in this study.

MBCs	ER status	PR status	HER2 status	germline pathogenic variants	LOH of germline pathogenic variants (LOH/NO LOH)	Clinically actionable variants in BC according OncoKb database	TMB (high/low)	MSI (high/low)
**#1**	Negative	Positive	NA	*PALB2* c.1984 A>T; p.Lys662Ter	LOH	–	High	NA
**#2**	Positive	Positive	Negative	–	n.e.	*PIK3CA* c.3145G>C; p.Gly1049Arg	Low	High
**#3**	Positive	Positive	Negative	–	n.e.	*PIK3CA* c.3140A>G; p.His1047Arg	High	Low
**#4**	Negative	Negative	Positive	*BRCA1* c.5266dup; p.Gln1756fs	NO LOH	–	Low	Low
**#5**	Positive	Positive	Negative	*BRCA1* c.4484G>T; p.Arg1495Met	LOH	–	Low	Low
**#6**	Positive	Positive	Negative	–	n.e.	–	Low	Low
**#7**	Positive	Positive	Positive	–	n.e.	*PIK3CA* c.3140A>T; p.His1047Leu	Low	Low
**#8**	Positive	Positive	Negative	–	n.e.	–	Low	High
**#9**	Positive	Positive	Negative	–	n.e.	–	Low	Low
**#10**	Positive	Positive	Negative	–	n.e.	*PIK3CA* c.1035T>A; p.Asn345Lys	Low	Low
**#11**	Positive	Positive	Negative	–	n.e.	*PIK3CA* c.1633G>A; p.Glu545Lys	Low	Low
**#12**	Positive	Positive	Positive	*BRCA2* c.6275_6276del; p.Leu2092fs	NO LOH	–	Low	Low
**#13**	Positive	Positive	Negative	–	n.e.	–	Low	Low
**#14**	Positive	Positive	Negative	–	n.e.	–	Low	Low
**#15**	Positive	Positive	Negative	–	n.e.	*PIK3CA* c.3140A>; p.His1047Arg	Low	Low

LOH, Loss Of Heterozygosity; NA, not available; n.e., not evaluated.

**Figure 2:**
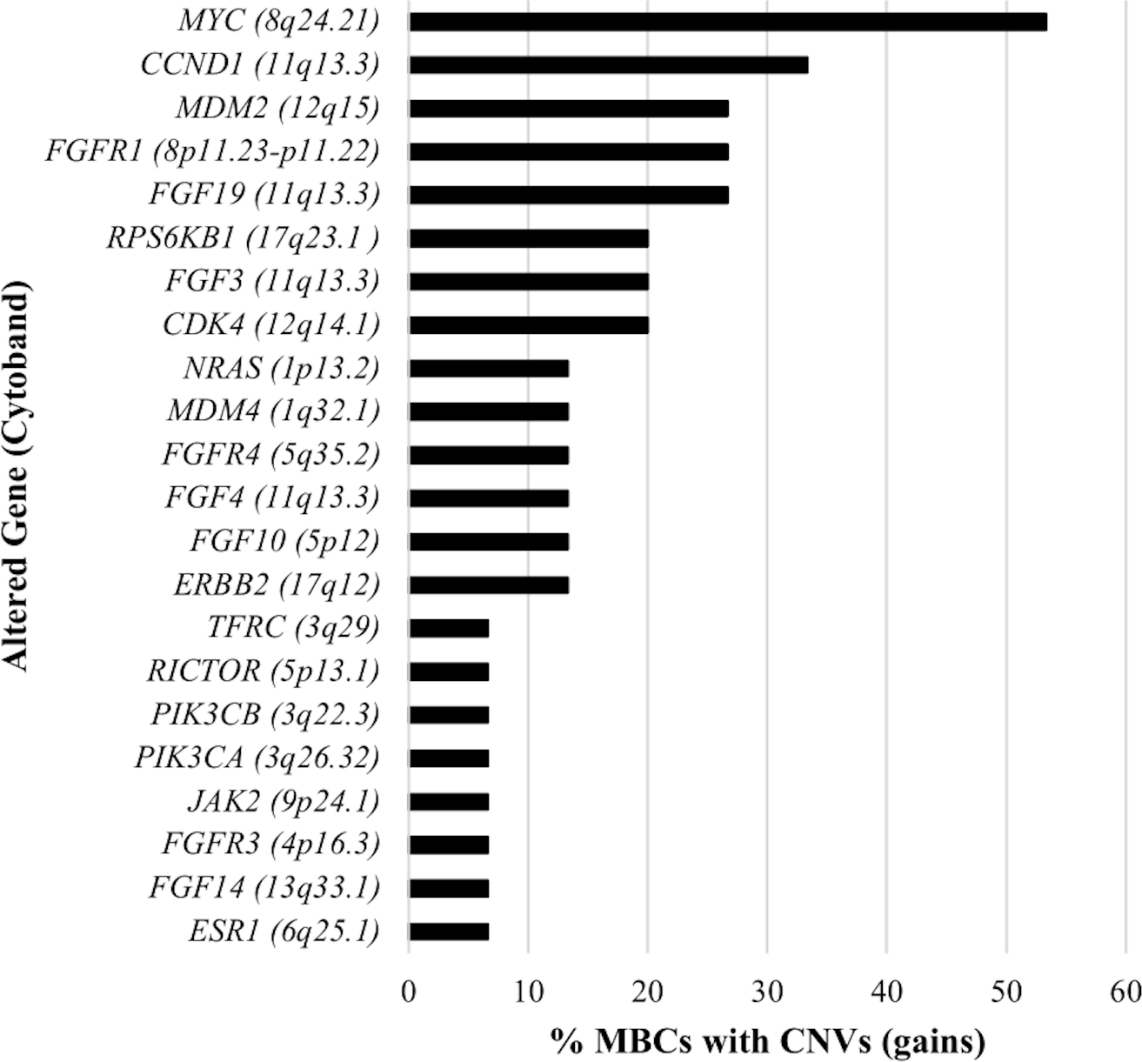
Percentage of MBCs with DNA Copy Number Variations (CNVs), by altered gene.

A total of 54 CNVs, all copy number gains, were detected in 22 genes in 13 (86.7%) MBCs (**Figure 2**). No copy number losses were found. CNVs most frequently detected were amplifications of 8q, 11q, and 12q cytobands. Specifically, *MYC* (8q24.21 cytoband) amplification was observed in eight (53.3%) MBCs with fold-change values ranging from 1.8 to 3.8 (median 1.9); *CCND1*, *FGF19, FGF3*, and *FGF4* (11q13.3 cytoband) amplification was observed in five (33.3%), four (26.7%), three (20.0%), and two (13.3%) MBCs, with median fold-change values of 3.7, 2.4, 2.9 and 5.9, respectively; *CDK4* (12q14.1 cytoband) and *MDM2* (12q15 cytoband) amplifications were observed in three (20.0%) and four (26.7%) MBCs, respectively, with median fold-change value of 2.9, each (**Supplementary Table 4**).

**Figure 3:**
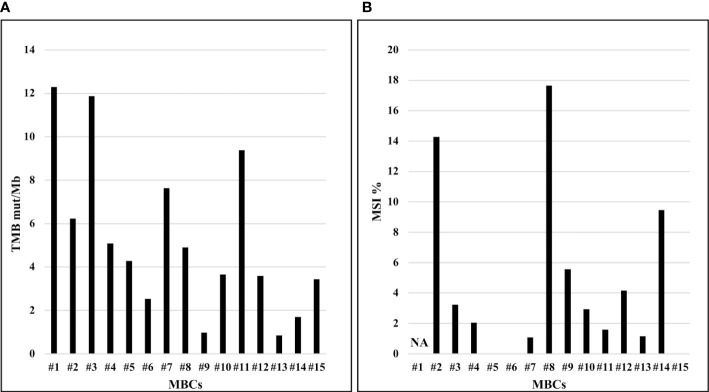
TMB (mut/Mb) and MSI (%) of MBCs analyzed. **(A)** Distribution of TMB (y-axis), by sample. **(B)** Distribution of MSI (y-axis), by sample. NA, not available.

The median TMB value was of 4.3 mut/Mb (range, 0.8‐12.3 mut/Mb) across all MBCs. High-TMB, was reported in two (13.3%) MBCs with TMB values of 12.3 mut/Mb and 11.9 mut/Mb, respectively (**Figure 3A**). The median percentage of MSI across all MBCs was 2.4% (range 0.0-17.6%). High-MSI was reported in two (13.3%) MBCs with MSI value of 17.6% and 14.3%, respectively (**Figure 3B**).

**Figure 4:**
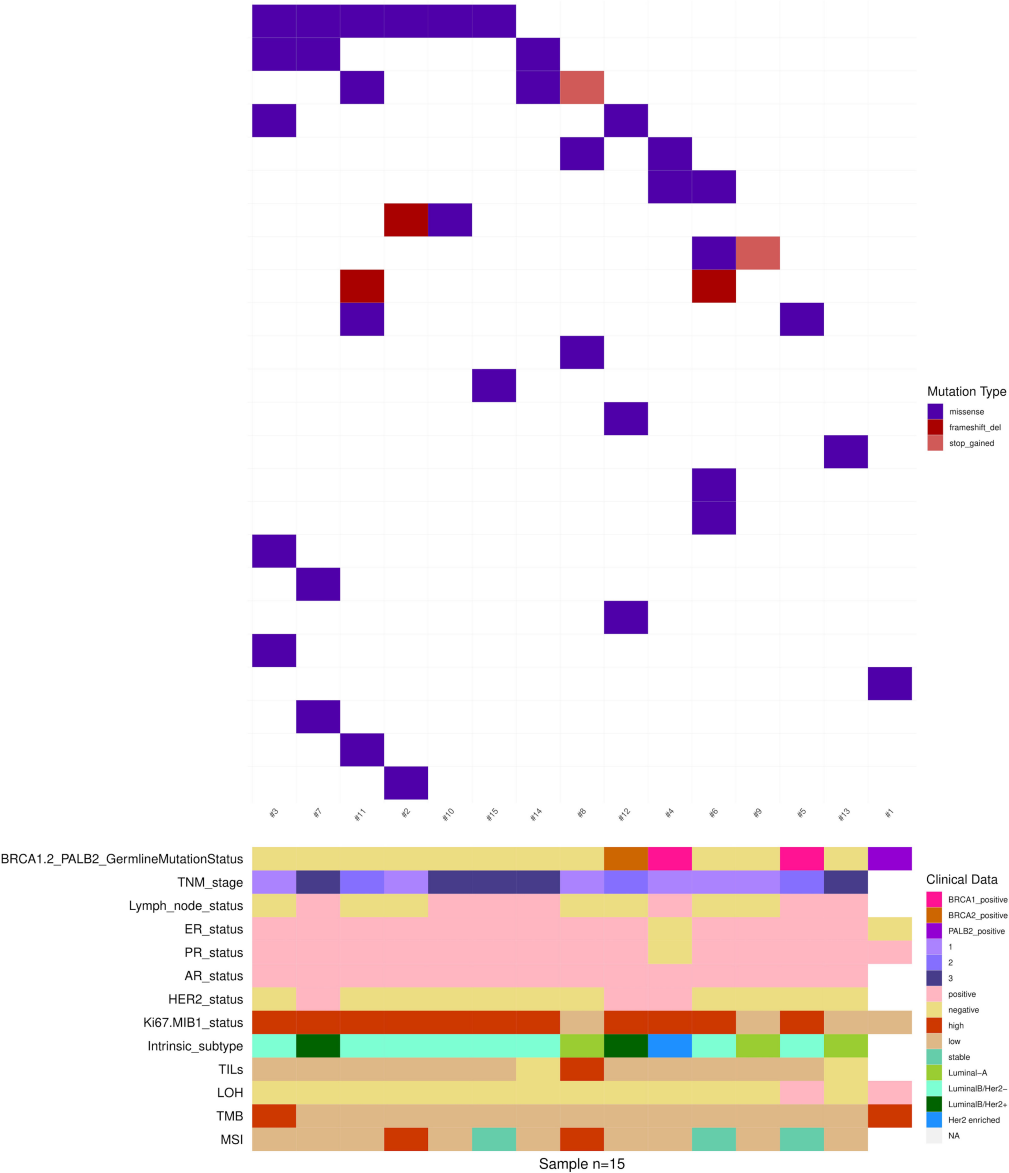
Somatic non-synonymous variants in 15 MBCs. Mutated genes are represented as rows, and individual patients are represented as columns. Clinical data, tumor histology and molecular biomarkers investigated are indicated by the horizontal bars.

For each MBC case, somatic alterations together with the main clinical-pathologic characteristics are depicted in **Figure 4**.

### Clinically significant variants

The OncoKB database was interrogated to evaluate whether the detected alterations were actionable targets in BC. **Table 2** shows the actionable molecular alterations identified in each MBC case together with the main clinical-pathologic characteristics.

According to OncoKB, at least one clinically significant somatic alteration was detected in 11 (73.3%) MBCs. Specifically, actionable targets included *PIK3CA* somatic variants, high-TMB and high-MSI.

Five BC specific *PIK3CA* alterations (c.1035T>A, p.Asn345Lys; c.1633G>A, p.Glu545Lys; c.3140A>G, p.His1047Arg; c.3140A>T, p.His1047Leu; c.3145G>C, p.Gly1049Arg), reported as predictors of response to PI3Kα-specific inhibitor combined with estrogen receptor antagonist, were identified in six (40%) MBCs (**Table 2**).

Two MBCs had high-TMB and two high-MSI. High-TMB and high-MSI phenotypes seem to associate with response to immune checkpoint inhibitors in several solid tumor types, including BC. Notably, one MBC case with high-TMB and one with high-MSI had also a *PIK3CA* somatic variant (**Table 2**).

As described above, four MBC cases harbored *BRCA1/2* and *PALB2* germline pathogenic variants and LOH occurred in two of them. According to OncoKB, *BRCA1/2* and *PALB2* pathogenic variants, with or without biallelic loss, predict response to PARP inhibitors. Notably, the MBC case with biallelic loss of *PALB2* was also classified as high-TMB (**Table 2**).

## Discussion

In this study, we characterized the molecular profiling of 15 MBC cases by large multigene panel sequencing, targeting 1.94 Mb of the genome at 523 cancer-relevant genes. To the best of our knowledge, at present only two studies explored the genomic landscape of MBC (10, 12).

The molecular landscape of our series, although relatively small, resembles and recapitulates the main molecular profiles of MBC as described so far.

In line with previous studies, we identified *PIK3CA* as the most frequently mutated gene in MBC. The *PIK3CA* somatic variant frequency in our MBC series was slightly higher than that reported by Piscuoglio et al. and Moelans et al. (10, 12), most likely because of differences in size or composition of the series examined. Overall, our results add to previous findings (18) highlighting the relevant role of *PIK3CA* alterations in MBC.

On the other hand, in line with previous studies (10, 12, 18, 19), our findings showed that *TP53* somatic variants are rare in MBC. *TP53* variants are frequently observed in FBCs, particularly with luminal B and triple negative subtypes (10, 12). The low frequency of these subtypes in MBCs might explain the low *TP53* variant frequency observed.

In addition, pathogenic variants in other genes were identified at low frequencies in our series, highlighting MBC heterogeneity. Among these, *ARID1A* pathogenic variants might be promising therapeutic targets in BC. *ARID1A* deficiency due to somatic mutations have been associated with impaired DNA damage repair in BC, thus prompting the development of synthetic lethality-based therapeutic strategies for ARID1A-mutated neoplasms (20, 21).

In this study, CNVs were observed in about 87% of MBCs and all were gene copy number gains. These findings are consistent with previous evidence showing that genomic gains are more common in MBCs while genomic losses are less frequent (22–27).

In line with previous data (10), our results showed that copy gains of *MYC* (8q24.21 cytoband), *CCND1* (11q13.3 cytoband) and *MDM2* (12q15 cytoband) genes were frequent in MBCs.

*MYC* and *CCND1* overexpression has been associated with resistance to endocrine therapy in BC (28, 29). Thus, our findings could be particularly relevant in the clinical management of MBC as endocrine therapy is most commonly used in MBC treatment (4).

*MDM2* gain has been shown to significantly correlate with a worse survival of Luminal BC patients allowing a further stratification of Luminal BC based on *MDM2* status (30). As Luminal is the most frequent subtype in MBCs, our findings may suggest that *MDM2* amplification might be investigated as prognostic biomarker.

The gene panel design allowed for the evaluation of comprehensive molecular biomarkers, including TMB and MSI, by NGS approach. To the best of our knowledge, the evaluation of TMB and MSI has not yet been performed in MBCs. In our series, only two MBCs showed high-TMB and two high-MSI, while the majority of cases showed low-TMB and low-MSI. These results are in line with data showing that high-TMB and high-MSI phenotypes are rarely observed in FBCs (31, 32).

A specific aim of this study was to characterize clinically actionable somatic alterations that may represent new predictive biomarkers for MBC patients. Our findings showed that NGS multigene panel sequencing can allow for the identification of MBC patients eligible for precision/targeted therapy. In our series about 73% of MBCs showed molecular alterations useful for more individualized therapeutic options, besides treatments commonly used in MBC patients based on hormonal and HER2 status (4). Our results are consistent with data from FBC reporting actionable molecular alterations in about 80% of BCs (33).

According to OncoKB database, actionable targets identified in our study include alterations in *BRCA1/2*, *PALB2, PIK3CA*, high-TMB and high-MSI.

It is known that cells with loss of *BRCA1/2* or *PALB2* function have sensitivity to PARP inhibitors (34–36). In this series, four MBC cases harbored *BRCA1/2* and *PALB2* germline pathogenic variants and LOH occurred in two of them. According to OncoKB, *BRCA1/2* and *PALB2* pathogenic variants, with or without bi-allelic loss, predict response to PARP inhibitors.

The PI3K-AKT-mTOR signaling pathway plays an important role in the development of BC (37) and in driving endocrine resistance (38). Targeting the PI3K-AKT-mTOR signaling pathway has become a promising therapeutic option in BC treatment (39). Somatic variants at codons 542, 545 and 1047 of *PIK3CA* gene have been demonstrated to be responsive to Alpelisib, a phosphatidylinositol-3-kinase inhibitor (40, 41). Alpelisib, in combination with Fulvestrant, an ER antagonist, is now FDA-approved for patients *PIK3CA*-mutated, HR-positive, HER2- negative BC after endocrine therapy-based treatments or with disease progression (42). In our series, five (40%) MBC cases showed *PIK3CA* alterations reported as predictors of response to PI3Kα-specific inhibitor combined with estrogen receptor antagonist.

High-TMB values in solid tumors are associated with response to immune checkpoint inhibitors (43). In our series, the MBC case with the highest TMB-value showed bi-allelic *PALB2* alteration (germline pathogenic variant and LOH). These findings are consistent with the hypothesis that *PALB2*-mutated BCs may be associated with high mutational load (44) and that the DNA repair genes inactivation may give rise to BC specific immune-phenotype, that could be leveraged with checkpoint blockade (45). Notably, the *PALB2*-mutated case was an ER-negative BC, a very rare occurrence in MBC, and some studies reported higher TMB values in ER-negative compared with ER-positive FBCs (46, 47). Further studies are needed to investigate possible associations between TMB status and clinical-pathologic variables in MBC.

While the clinical management of MSI phenotype is well-established in colorectal and endometrial cancers, the predictive value of MSI status in BC is not well-know and this is most likely due to the low frequency (1-2%) of MSI phenotype in BC (48, 49). In our series two MBCs showed a high-MSI phenotype. Specifically, one was a Luminal B/HER2 negative BC with high proliferation activity and the other a Luminal A with low proliferation activity. These results are in line with literature data showing that MSI phenotype can be detected across different BC subtypes (50),

It is interesting to note that all MBCs with a stable MSI also showed low-TMB and that the cases with high-MSI do not correspond to the cases with high-TMB, thus in line with findings suggesting that MSI is not necessary for high-TMB (51).

Despite the low prevalence, the MSI phenotype BC was shown to be highly responsive to immunotherapy with checkpoint inhibitors (52). Thus, our findings suggest that the determination of MSI phenotype as a biomarker of response to immune checkpoint inhibitors, potentially combined with TMB, could be crucial to better identify eligible MBC patients for this therapeutic approach. Further insights on the molecular profiles associated with TMB and MSI status in MBC cases with and without germline pathogenic variants in genes involved in genome stability may provide a better characterization of MBC somatic landscape and lead to a more accurate classification of MBC molecular subtypes, with potential therapeutic implications.

This study has a few limitations. First, it is a pilot, retrospective study on a small cohort of patients; thus, the occurrence of possible bias related to small numbers for the observed variant frequencies cannot be excluded and may affect the findings. Second, the variability in current computational methodologies for the identification of pathogenic somatic variants and the estimation of TMB represent a challenge. Our pipeline, whose results are shown here, is based on evidence from recent methodological comparisons (14, 15, 53). However, there is a need for standardization and harmonization of assessment methodologies and parameters across studies. For example, there is currently no consensus on the minimum variant allele frequency and total read depth to be considered for variant selection. Some studies highlight the importance of using an allelic frequency threshold of 5% (54–56). In our study, only two of the 40 somatic variants identified showed an allelic frequency <10.0% and a good read depth (>100). Foundation Medicine FoundationOne CDx panel is the only current NGS test approved by FDA to measure TMB (57). In this study, we used TruSight Oncology 500 Panel, since its size and gene composition allowed for both a comprehensive exploration of genomic landscape and the evaluation of molecular biomarkers such as TMB and MSI. Further studies including a larger number of MBCs are needed to validate our molecular findings and the computational methodologies proposed here.

## Conclusions

Although MBC peculiarities need to be further investigated at genetic and molecular level, our results suggest that the use of targeted gene panel sequencing in clinical practice may represents a fundamental step in the improvement of MBC management. The identification of actionable molecular alterations may concur to establish, with greater precision, which MBC patients can benefit from new therapeutic strategies as well as being useful for the development of new drugs.

In conclusion, this study adds new data to the actionable genomic landscape of MBC highlighting the importance of the incorporation of NGS testing in the clinical management of MBC eventually leading to the implementation of precision medicine for MBC patients.

## Data availability statement

The datasets presented in this study can be found in online repositories. The names of the repository/repositories and accession number(s) can be found below: European Nucleotide Archive PRJEB57620.

## Author contributions

VV, VS, and MK analyzed genomic data. VV, VS, MK, LdF and LO interpreted the results. VV wrote the manuscript. VS, AB, GC, MK, LdF, BC, AC, GdA, GG and LO revised the manuscript and contributed to the language editing of the manuscript. VV, GP, SM, BC, MGP, AN, AC, GdA, GG and LO collected the clinical pathologic characteristics. GdA, BC, MGP performed and interpreted immunohistochemistry analyses. SM, AC, Gd and LO conceived, designed, and planned the study. All authors contributed to the article and approved the submitted version.
