# Supplementary material for: Molecular profiling of male breast cancer by multigene panel testing: Implications for precision oncology
Source: Front Oncol. 2023 Jan 6;12:1092201. doi: 10.3389/fonc.2022.1092201 (PMC9854133; doi:10.3389/fonc.2022.1092201)
Supplement: Supplementary file 1 [file DataSheet_1.docx]

***Sequencing data and DNA quality metrics***

After the DNA extraction from FFPE slides, the quality control metric of samples for sequencing was determined by the deltaCycle threshold (dCt) that must be under 5. Study samples had a median dCt of 2.49 (range 1.0-8.1). Despite the maximum dCt run was 8.1, samples were all sequenced to determine the validity of this measure in selecting samples for sequencing.

Regarding the DNA sequencing metrics, the median percentage of exon covered at least 50X was 98.2% (range 75.2-99.5%), the median insert size was 113 bp (range 77-127 bp), and the median exon coverage was 164 counts (range 70-315 counts). Regarding other DNA expanded metrics, the median reads per sample was 126758812 (range 70182822-156975212 reads), the median percentage of read enrichment and of chimeric reads were 75.8% (range 69.6-82.6%) and 3.2% (range 1.7-5.1%), respectively. Moreover, at the end of variant annotation step, we identified a median of 117248 (range 68359-224837) variants and a median of 3 (range 0-10) Copy Number Variations (CNVs) for the series analyzed. Specifically, we identified a median number of 1225 (range 1128-1314) variants and a median number of 3 (range 0-10) CNVs that passed the quality filters and marked as PASS in the output file. Starting from these PASS variants, we applied the variant filtering step to perform the somatic variant and TMB calling. The number of total and PASS variants and CNV identified for each sample analyzed is shown in **Supplementary Table 2.**
